# Book Reviews

**Published:** 1872-07-15

**Authors:** 


### Book Reviews.

Catalogue of the Library of the Surgeon General's Office, United States Army; with an Alphabetical Index of Subjects.

This is a large and handsomely bound volume of 454 pages, giving the titles of 13,330 volumes. The alphabetical index of subjects although not complete, will be found very valuable for reference.

The library of the Surgeon General's Office dates only from the commencement of the late war, at which time it consisted of about 350 volumes of text books and journals. Since that time the increase by donation, exchange and purchase, has been very rapid.

That there is need in this country of a medical library of this character is sufficiently evident from the fact that in all the public medical libraries of the Unite States put together, it would not be possible to verify from the original authorities the references given by standard English or German authors. No complete collection of American medical literature is in existance; and the most complete in this country is in private hands, and not accessible to the public; while every year adds to the difficulty of forming such a collection as the government should possess. The books are now safely and conveniently arranged in the fire-proof buildings of the Army Medical Museum, and are accessible to the public under rules and regulations essentially the same as those for the Library of Congress.

There is also a pamphlet supplement accompanying the catalogue, giving a list of the American Medical Journals, and the numbers which are wanting to complete the files of the Library.

Memoranda on Poisons, by the late Thomas Hawkes Tanner, M.D. F. L. S.: Lindsay & Blakiston, Philadelphia. Price 75 cts.

This is a little pocket volume of 150 pages, completely remodeled from its former editions. With a view to making it more valuable, especially to the student, the chemical bearings of poisoning are carefully considered, and the more important and reliable tests are in each instance given, as well as the more important processes for separating poisons from organic admixtures.

Dr. Rigby's Obstetric Memoranda. Fourth Edition; Revised and Enlarged by Alfred Medows, M. D. Philadelphia: Lindsay & Blakiston. Price 50 cts.

This little pocket memorandum book is already well known through its former editions. The changes made in the present edition are sufficiently explained in the following extract from the preface: "The fact that this little book was originally written by the late Dr. Rigby, and that three large editions have been exhausted, is sufficient to justify the issue of a new edition which I have had pleasure in revising. I have ventured to alter the arrangement in some important particulars, to add some new matter, and to omit some of the old, hoping thereby to further the original intention of the author, by making the book as practically useful as possible."

